# Urban Greening Effect on Land Surface Temperature

**DOI:** 10.3390/s22114168

**Published:** 2022-05-30

**Authors:** Anita Zaitunah, Samsuri, Angelia Frecella Silitonga, Lailan Syaufina

**Affiliations:** 1 Faculty of Forestry, Universitas Sumatera Utara, Jalan Tri Dharma Ujung No 1, Medan 20155, North Sumatra, Indonesia; samsuri@usu.ac.id (S.); angeliasilitonga3@gmail.com (A.F.S.); 2 JATI—Sumatran Forestry Analysis Study Center, Medan 20155, North Sumatra, Indonesia; 3 Department of Silviculture, Faculty of Forestry and Environment, Bogor Agricultural University, Bogor 16680, West Java, Indonesia; lailans@apps.ipb.ac.id

**Keywords:** city park, LST, NDVI, remote sensing, urban greening

## Abstract

Urbanization has accelerated the conversion of vegetated land to built-up regions. The purpose of this study was to evaluate the effects of urban park configuration on the Land Surface Temperature of the park and adjacent areas. In urban parks, the study analyzed the Normalized Difference Vegetation Index (NDVI), the Normalized Difference Built-up Index (NDBI), and the Land Surface Temperature (LST). The NDVI categorization process resulted in the development of a vegetation density distribution. The majority of Medan’s urban areas were categorized as low density, as seen by their low NDVI values. The NDBI values were significantly higher in the majority of the area. This shows that the majority of places are experiencing a decline in vegetation cover. The density of vegetation varies according to the placement of park components such as trees, mixed plants, recreation, and sports areas. According to LST data, the temperature in the urban park was cooler than in the surrounding areas. Although the surrounding areas are densely populated, urban parks are dominated by trees. Additionally, there is a green space adjacent to the park, which is a green lane that runs alongside the main roadways.

## 1. Introduction

In the next century, approximately 60% of the world’s population will be living in cities [1]. Urbanization alters the water cycle, deteriorates water quality, and damages habitats [2]. The effects of climate change, urbanization, and population increase in urban zones include heat waves, droughts, air pollution, and other environmental problems. Climate change necessitates a significant reduction in energy demand and greenhouse gas emissions. Aside from that, cities must adapt to meet the problems posed by climate change [3]. Urban green spaces’ ecosystem services can help mitigate the effects of these issues [4]. The composition and existence of vegetation affected the microclimate of the urban and surrounding areas [5,6].

Effective communication between scientists, planners, managers, and users of urban green spaces may be critical to the success of urban biodiversity conservation [7]. A holistic approach is required to improve urban resilience based on green infrastructure by addressing a wider variety of ecosystem disturbances and disasters to produce outcomes that develop urban expansion’s environmental and ecological benefits [8]. Urban parks can help urban people’s physical and psychological health and society’s social, economic, and environmental well-being [9]. Noise and air pollution are minimized by the park and street trees. They promote fitness, relaxation, and socialization. It may be difficult to supply these services during extreme weather events such as heat waves and drought [3]. A park could provide a spot for individuals to escape the heat. Urban parks are considered a natural solution to the city’s many environmental issues [1].

Urban parks provide benefits to the community and environmental quality. With over half of the world’s population residing in cities, urban parks are critical sites for human–nature interactions, and their vegetation can provide a variety of ecosystem services [10]. Urban green spaces, such as parks, are becoming more critical as cities densify. Urban parks offer physical and social activity, recreation, and relaxation, all of which contribute to a person’s overall well-being [11].

Sixty percent of the land that will be urbanized by 2030 remains undeveloped. Contemporary urban expansion is unsustainable, emphasizing density, frequently at the price of urban green space, even as our awareness of the linkages between green spaces and human well-being, particularly health, continues to grow [12]. Urban parks are a vital component of public green space and a popular gathering place for city people. The outdoor thermal comfort of people visiting and staying in urban parks is important in determining the urban environment’s responsibility [13]. City parks provide some habitat [14]. Ecosystem services vary in type and quantity within a park based on vegetation [1]. As an illustration of the bee conservation potential of urban green places, a large and diversified city park can provide an optimal home for bees [15] and Avian species [16].

City parks, as vital spaces designed for physical activity, are key in preventing chronic diseases and boosting public health [17]. The health advantages of urban parks have received a lot of attention recently. Observing urban parks has a calming effect on physiological and psychological systems [18]. Urban parks can contribute to older persons’ physical, social, and psychological well-being [19]. While park vegetation is often viewed as a beneficial method of reducing particle pollution in urban areas, there are no quantitative data on its efficiency [20].

Medan, as one of the largest cities in Indonesia, has grown to be one of the characteristics of large cities. An increase in housing demand has accompanied population growth. The economic activities of a citizen also contribute to the city’s development. Buildings for these activities have been constructed in the city’s central business district and neighboring areas. Bare land and vegetated land have been converted into built-up areas. The existence of open space is critical to society’s dynamics. Open space is defined as a location for the growth of individual and collective human life and a place for the survival and sustainable development of other living species [21].

Remote sensing could aid in identifying and resolving a city’s environmental issues. Normalized Difference Vegetation Index (NDVI) and Normalized Difference Built-up Index (NDBI) can provide a spatial overview of a city’s vegetation and built-up areas. The vegetation index is one of the parameters used to assess an area’s vegetation state. Land Surface Temperature (LST) may be used to demonstrate the effect of vegetation on surface temperature. NDVI was one of the first remote sensing analytical tools used to simplify the intricacies of multispectral imagery and is today the most widely used index for assessing vegetation [22]. It can be used to determine one sort of vegetation density. This approach makes it suitable for analyzing vegetation conditions. Remote sensing technology can be used to gather data on the vegetation density, the size of the land area, and field conditions.

This study aimed to assess how the configuration of city park components relates to vegetation density and surface temperature. Each park has its layout, composition, and distribution of vegetation and its service and support sections. The study on vegetation density in the urban park and the surrounding built-up areas may provide insight into the presence of vegetation within certain areas, which is useful for creating a high-quality urban environment. The information should also aid the government in enhancing the quality of city parks and supporting the need for green space. The study on vegetation density and Land Surface Temperature may provide insight into the importance of the presence of vegetation within specific areas to create and maintain a high-quality urban environment.

## 2. Materials and Methods

**Figure 1:**
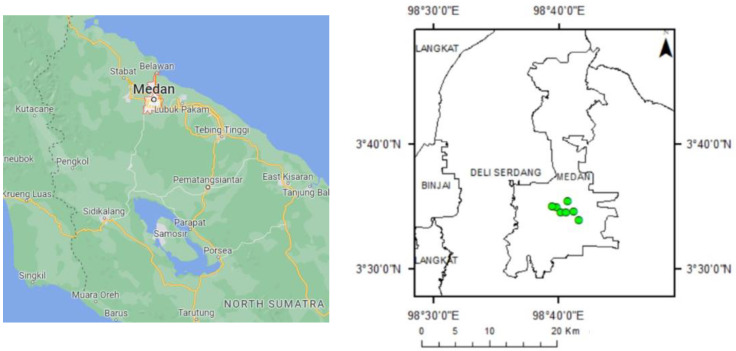
Research Site Map (the green symbols indicate the location of the studied urban parks). (Source: Google map (left)).

This study was taken place between November 2020 and March 2021 at Medan City (Figure 1). The analysis was conducted in Forest Management Laboratory, Faculty of Forestry, University of Sumatera Utara.

Medan lies between 3°27′–3°47′ of North Latitude 98°35′–98°44′ of East Longitude with a height above sea level 2.5–37.5 m. Medan Municipality is one of the regencies/municipalities in North Sumatera Province, with about 265.10 km^2^. This municipality is the center of government of North Sumatera Province which is boundaries by regency of Deli Serdang in the north, south, west, and east. Medan had a tropical climate in 2019. The minimum temperature was 21 °C, and the maximum temperature was 36 °C. As part of a city, there are parks and other green spaces. Some parks in Medan are devoted to preserving environmental quality and providing public service for enjoyment and health enhancement.

Six parks were identified in this investigation. The parks include Merdeka Square, Ahmad Yani, Teladan, Sri Deli, Gajah Mada, and Petula (Table 1). These parks serve the same purpose as green open spaces by actively supporting the recreational and sporting needs of the public. Each park has a diversity of land cover, including tree-dominated green space and sports open space.

### 2.1. Data Preparation

The data retrieval and analysis tools utilized in this investigation are described below. Global Positioning System (GPS) and digital cameras are used to retrieve field data. The data for this study include satellite image, administration map, and ground check data. Landsat-8 OLI image of year 2019 was used as the primary data source for analysis using ArcGis 10.3 (Esri, Indonesia). The date of acquisition of the image was 11 August 2019. Additionally, Google Earth image was used as supporting data to create a more detailed visual media. The administration map of Medan was used to delimit the study region.

In this study, both primary and secondary data were collected. Primary data are gathered through direct observation (field check) utilizing a method of purposive sampling. Image band merging, correction, and cropping are the data preparation stages. Field surveys are conducted to directly assess the field conditions and verify the information generated by image processing using NDVI transformation.

### 2.2. Normalized Difference Vegetation Index and Vegetation Density Analysis

The NDVI transformation is performed using the red and near infrared bands, bands 4 (red) and 5 (near infrared) for Landsat-8 OLI. The NDVI works based on measuring the rate of greening. With a range of values between −1 and +1, this NDVI has a range of performance on land usage. Cloud, water, and non-vegetation objects have an NDVI value less than zero. The higher the NDVI number, the higher the vegetation density; conversely, the lower the value, the lower the vegetation density in the area. The vegetation density level is classified into five classes based on the NDVI value and land cover: non-vegetation, low, moderate, dense, and high-density classes. The area was classified into vegetation density classes based on field observations of land cover, vegetation presence, and considering some references [23,24,25]. Each class has a range of NDVI values.

### 2.3. Normalized Difference Built-Up Index

Normalized Difference Built-up Index (NDBI) is the linear combination of near infrared (NIR) band (0.76~0.90 μm) and the middle infrared (MIR) band (1.55~1.75 μm). In comparison to other land use/land cover surfaces, built-up lands exhibit a higher reflectance in the MIR wavelength range than in the NIR wavelength range (0.76–0.90 m). The MIR corresponds to Landsat-8 band 6, and NIR corresponds to Landsat-8 band 5. The equation of NDBI is explained in Malik et al. [26].

### 2.4. Land Surface Temperature Analysis

Land Surface Temperature analysis includes some processing stages. The stages of LST processing are conversion of the spectral radiance value into brightness temperature, classification of NDVI (Normalized Difference Vegetation Index), determination of the value of proportion of vegetation, and emissivity value (ε), Land Surface Temperature (LST). The LST temperature is the earth’s surface temperature at its point of contact with the atmosphere. Several mathematical algorithms were employed and processed in ArcGIS to determine LST using Landsat-8 satellite data. There are several steps involved in evaluating LST derived from Landsat-8 data. These steps include converting the digital number of a satellite to radiance, calculating the brightness temperature, and converting the brightness temperature to LST, which requires the emissivity calculated from the proportion of vegetation [26].

### 2.5. Urban Park Analysis

The observation was conducted to find out the presence of vegetation. In this study, each park’s NDVI and LST analysis and classification of vegetation density were undertaken. The analysis’s findings may describe the parks’ state in terms of vegetation and non-vegetated areas and their relation to surface temperature. The analysis could serve as a guide for increasing the park’s quality as a critical component of urban green space.

## 3. Results

### 3.1. NDVI, NDBI and LST of Medan

**Figure 2:**
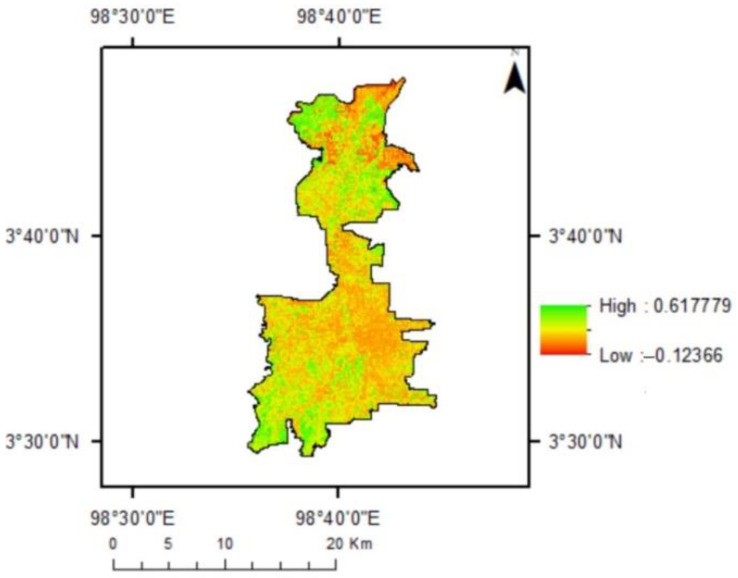
Map of NDVI Distribution in Medan City in 2019.

The NDVI analysis has confirmed the development of the urban areas of Medan. Figure 2 illustrates the distribution of NDVI values in Medan in 2019. Although most places had lower NDVI values, certain areas retained substantial vegetated areas, as verified by higher NDVI values.

Negative values correspond to locations with water, marshy surfaces, artificial structures, rocks, and clouds; bare soil often produces values between 0.1 and 0.2, whereas plants consistently have positive values between 0.2 and 1. The values are more than 0.5 for healthy, dense vegetation canopy, while the values range from 0.2 to 0.5 for sparse vegetation. NDVI readings are typically between 0.2 and 0.4 for places with minimal vegetation and 0.4 to 0.6 for areas with moderate vegetation, with anything above 0.6 indicating the highest potential green density [27]. The highest NDVI value in Medan was 0.618. A high value indicates the presence of high vegetation density. Some areas have negative values found in the field as watery areas in mangroves, paddy fields, and fishponds.

**Figure 3:**
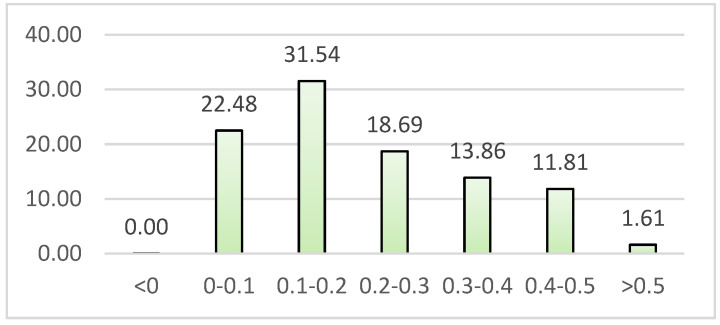
Distribution of NDVI Value in Medan in 2019. x NDVI values, y: percentage of areas (%).

The NDVI values in the year 2019 are depicted in Figure 3. Over 50% of the region had values less than 0.2. The fraction of locations with higher NDVI values decreased. Only 1.61 percent of the area had NDVI values greater than 0.5. This suggests that the majority of areas were devoid of vegetation.

**Figure 4:**
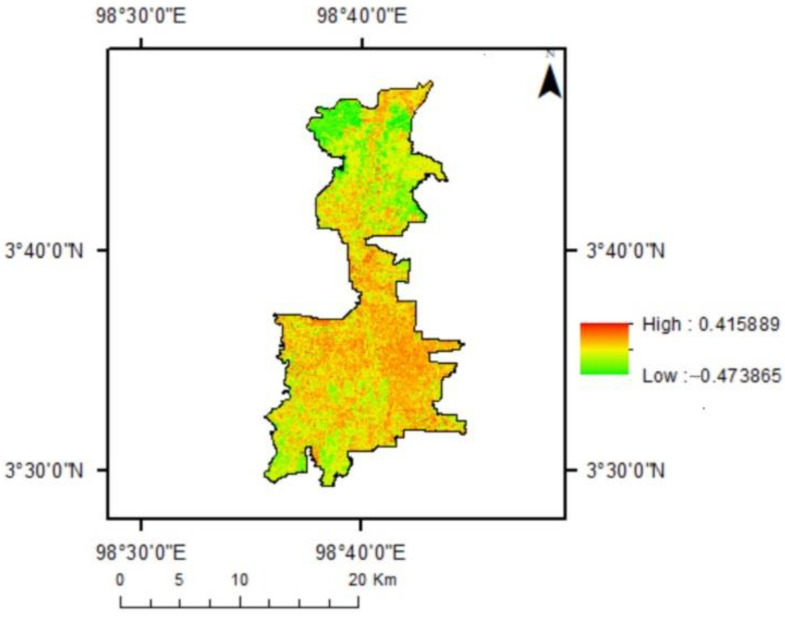
NDBI of Medan year 2019.

NDBI values range between −1 and 1. The bigger the NDBI, the greater the built-up area portion. Figure 4 shows most of the area in Medan was built-up areas represented by higher NDBI values (with red color). Some green spaces and water body found in the upper area of Medan. The site included mangroves, fish ponds, mixed gardens, and paddy fields.

**Figure 5:**
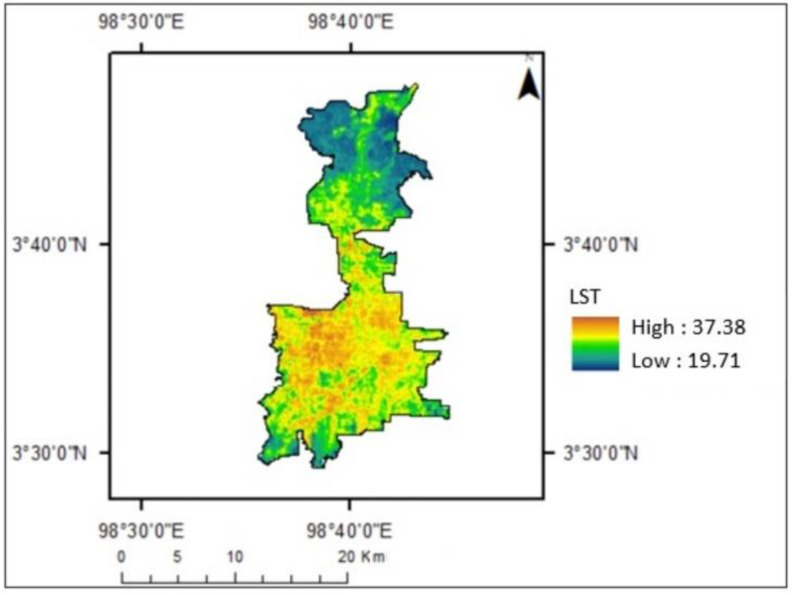
LST of Medan in 2019.

**Figure 6:**
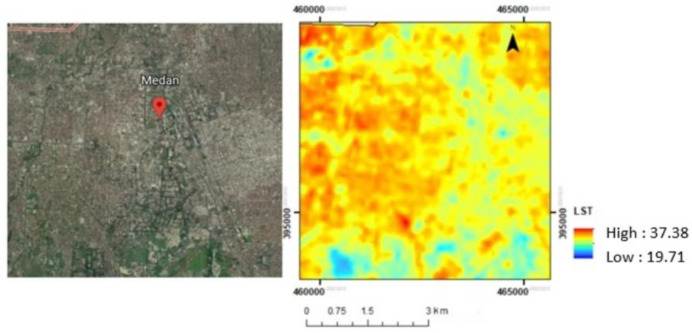
Visualization by Google Earth (**left**) and LST (**right**).

Figure 5 shows the variation of Land Surface Temperature within Medan city in 2019. The temperature range in Medan is between 19.7 and 32.38 °C. There was a lower temperature in the city’s outer ring and the upper part of the city, which is the location of mangrove and agricultural areas. In the city center, the temperature was higher than in the other areas. It was built-up areas including settlements and buildings. Figure 6 provides an overview of an image and LST of Medan.

### 3.2. Comparison of Park Characteristics

**Figure 7:**
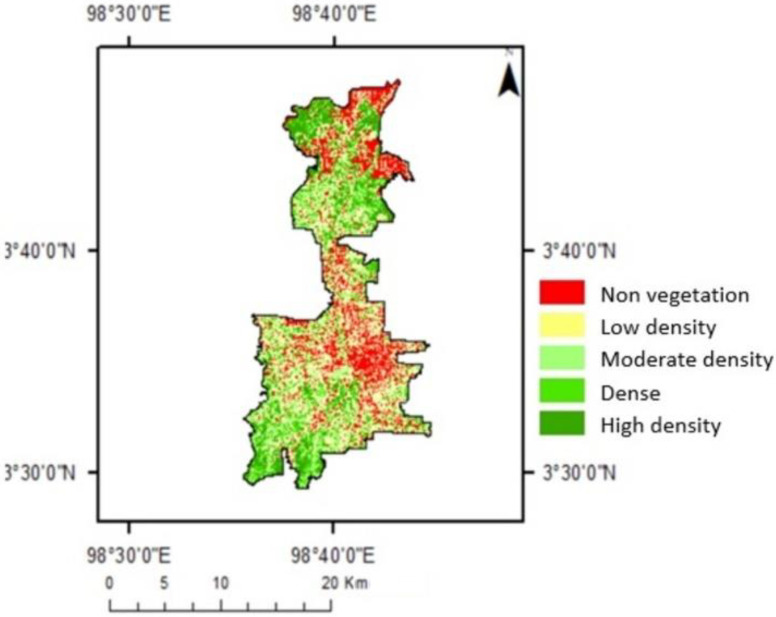
Map of vegetation density classes of Medan City in 2019.

Figure 7 shows vegetation density classes in the study area. The area with NDVI values below 0.1 was mainly built-up areas. The area with NDVI values between 0.1 and 0.2 is comprised of buildings, roads, villages, trees, and also grasses. In areas with NDVI values between 0.2 and 0.3, vegetation such as trees and grass can be found in the vicinity of buildings, roads, and settlements.

**Figure 8:**
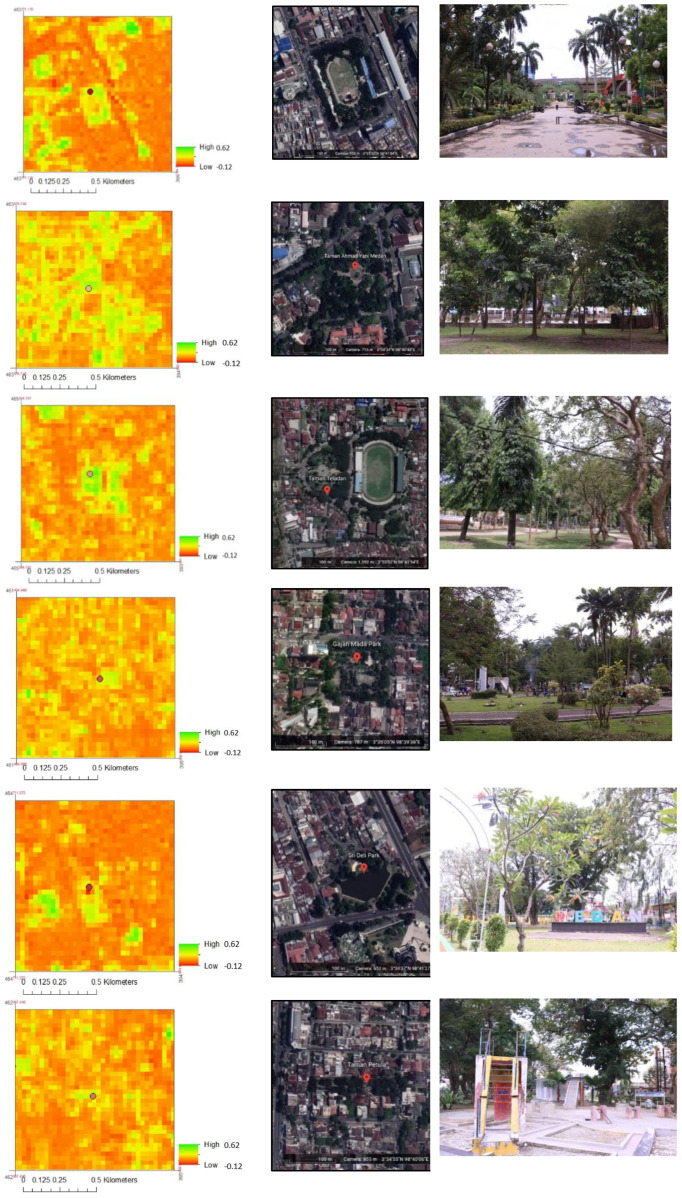
Parks’ overview in NDVI, Google Earth image, and in the field.

The dense class included city parks, oil palm plantations, mixed gardens, settlements, urban woods, and rice fields. Figure 8 shows the visualization of the area from the NDVI map, Google Earth image, and in the park. Those figures provide an overview of how differences in land cover components provide different results for NDVI values.

Figure 8 provides an overview of the presence of all parks in the city. There were different NDVI values within these parks. Medan Merdeka Park is located in the center of Medan. The greener area shows the higher NDVI values. The area with no vegetation shows the lowest values.

The observation of Ahmad Yani Park through the Google Earth image and field shows that the park has dense trees in most areas. In the surrounding areas, there are also trees along the main road. Buildings and settlements surround the park. Teladan Park has different characteristics compared to other parks. This park is in conjunction with a sports field. Some dense trees are part of the park. There is also an open area in this park. The park is in the middle of dense settlements. There are dense trees in Gajah Mada Park but also some open areas. In the surrounding areas, there are also trees along the main road. The park is surrounded by buildings and settlements. 

Sri Deli Park has different characteristics compared to other parks. There is a large garden pond. Some dense trees exist, but there is also an open area in this park. The park is close to the main road, surrounded by buildings and settlements. An overview of different NDVI values and the location of the park can be seen in Figure 8.

Figure 8 illustrates a difference in NDVI values in Petula Park. It is an elongated park. There are dense trees in the park but also some open areas. In the surrounding areas, there are also trees along the main road. The park is surrounded by buildings and settlements.

**Figure 9:**
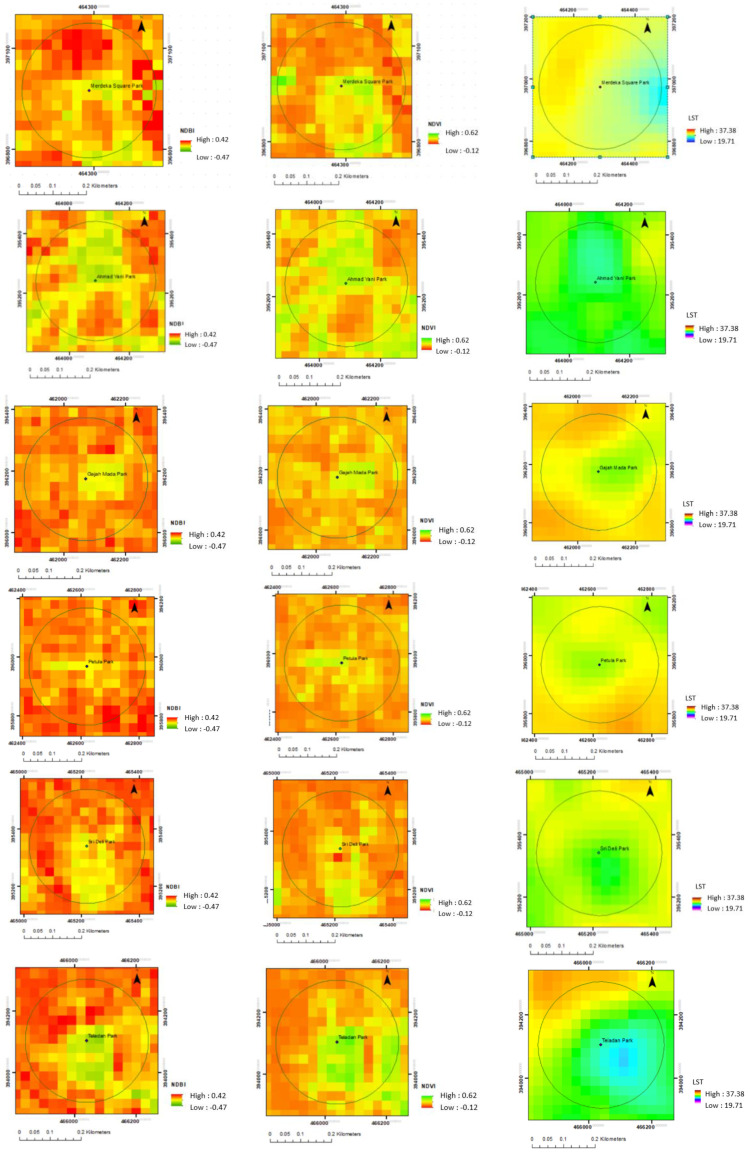
NDBI, NDVI and LST in Each Park in Medan.

Figure 9 shows differences in temperature within each park. There are some green spaces in the park. There are also trees in the surroundings, including trees along the road and trees in the home yard or around buildings. Based on the view of the Google Earth image and field observation, the area with vegetation shows a lower temperature.

Merdeka Square Park is located in the city center. In the center of the park is the large bare land. The vegetation includes mainly trees in the outer ring of the field. Figure 9 shows the area of the park and the surroundings. Higher NDVI, lower NDBI, and lower LST are shown in this area of the park. The area with vegetation shows higher NDVI and lower temperature.

Ahmad Yani Park has dense trees. There are also trees along the main road near the park. The park is surrounded by buildings and settlements. Figure 9 shows the area of the park and the surroundings. The area of the park that is dominated by trees shows a lower temperature than the surrounding area.

There are dense trees in the park but also some open areas within. In the surrounding areas, there are also trees along the main road. The park is surrounded by buildings and settlements. Figure 9 shows the lower NDBI and lower temperatures within the park and nearby areas. The park shows higher NDVI values compared to other nearby areas. The higher NDVI values indicate more vegetation in the area. The vegetated area in the park shows a lower temperature.

There are dense trees in Petula Park but also some open areas. In the surrounding areas, there are also trees along the main road. The park is surrounded by buildings and settlements. Figure 9 also shows that the main area of the park shows the lowest LST and NDBI but the highest NDVI. Most of the areas surrounding the park show lower NDVI and higher LST. Areas with vegetation show a higher value of NDVI and lower temperature.

As mentioned before, Sri Deli Park has different characteristics. There is a large garden pond. There is a variety of land cover within the park, including dense trees area and open spaces. Figure 9 shows the variation of NDBI, NDVI, and LST within and surrounding the park. The park shows lower LST and NDBI. The higher NDVI is indicated in the area with more vegetation. The vegetated area has a lower temperature.

Teladan Park is in conjunction with a sports field. There are dense trees in this park, yet there is also open space. The park is in the middle of dense settlements. However, there are also some green spaces nearby the sports field. Figure 9 shows that the lowest LST was in the main park areas. Higher NDBI is shown in the surrounding park area. Higher NDVI is shown in the park area, which was dominated by trees.

The analysis shows different values of NDVI, NDBI, and LST within the park and the surrounding areas. The values of NDVI, NDBI, and LST show the different pattern within vegetated areas and less or non-vegetated areas.

## 4. Discussion

### 4.1. Vegetation Role in Urban Park

A city park is an open space with social and aesthetic functions that is used for leisure activities, education, or other purposes. Environmental activities involve arranging a piece of land so that it is attractive to the owner or user, pleasant, and secure. These rules are used to classify each green open space division.

While urban parks may provide a variety of ecological services, community viewpoints can impact park conservation and biodiversity. Cultural ecosystem services, or the intangible advantages that people receive from nature, can encourage park use and urge local government to take action [28]. Urban green spaces provide vital recreational functions. In general, park visitors enjoy a mix of biotic, abiotic, and man-made park infrastructure features and attributes when they appreciate “the green” [29]. Trees play a significant role in determining the spatial characteristics of outdoor environments [30].

Vegetation density is one factor that determines vegetation’s appearance in an image. Vegetation density is commonly expressed as a percentage to indicate the level of vegetation density. The usage of land in a city is highly diverse, which results in a similarly diverse categorization. Vegetation analysis is a technique for determining the content of vegetation in the form of plant structures. Growth, stratification, and canopy closure are all components of vegetation structure. The vegetation index is a method developed in imaging (often multi-channel image) to highlight various characteristics of vegetation density or other densities, such as biomass, leaf area index, chlorophyll concentration, and so on. Indeed, this vegetation index is a mathematical modification that simultaneously utilizes many channels and generates a new image more representative of the vegetation phenomena.

Medan City Park is a public space that is open to all citizens. The park features a playground, a sports arena, a shower pool, a hall, and other recreational facilities that cater to people of all ages and genders. Additionally, the entire area of Medan city park features a children’s playground, and several parks provide areas for learning and discussion. In a municipal park, planting should be performed according to the type of plant. This might alter the shape and function of the garden.

The most comfortable circumstances were found in natural places with a multi-layered and dense canopy cover. However, if a plantation is excessively dense, the cooling efficiency of the vegetation may be reduced [31]. These characteristics must be present for a city park to attract the attention of city visitors. Tourist involvement is inextricably linked to the tourist and recreational design of parks. As a result, the issue of competent park design is critical to the city’s increased appeal [32]. The administrator of a city park must address a variety of park-related demands as a provider of social and ecological services necessary for the city’s sustainability, both profitable and non-profitable [33]. Urbanization and aging are the two fundamental social processes now influencing the world. In this context, especially in wealthy countries with the greatest rates of over-60s, establishing elder-friendly cities should be a priority [34].

State and local governments must implement park regulations or standards that serve as a foundation for designing a park system and establishing a minimum standard for city parks that include free, accessible, and safe physical activity spaces and sports facilities [17]. During a pandemic, urban parks and big outdoor, open spaces can provide citizens with a safe space for outdoor activities and social contact in a green environment and operate as a buffer zone to maintain good health and quality of life [35]. The importance of urban planners and landscape architects to optimize outdoor thermal comfort in metropolitan settings to create a more comfortable and healthier living environment for city dwellers [14]. As mentioned by [36], managers of urban parks are responsible for preserving the parks’ natural function and quality while also accommodating visitor preferences. Increased communication and collaboration among governmental agencies, non-profit organizations, and community members and continued investment in park management and interdisciplinary mixed methods research all have the potential to enhance urban parks’ numerous ecological and social benefits. Onose et al. [34] mentioned the needs, demands, and desires of elderly people in decision-making processes, aiming to create inclusive and senior-friendly parks.

### 4.2. Urban Park and Its Spatial Characteristics

Although there are different park characteristics, the park shows a similar spatial pattern. The park area shows lower LST and NDBI but higher NDVI compared to the surrounding areas. The main park area shows the lowest LST. It means the park area has lower temperatures than other areas. The nearby area has a lower temperature than the further area from the park. LST is also related to land cover classes of the urban areas [37].

Plants in public green spaces contribute significantly to cities’ environmental and aesthetic enhancement. Green spaces such as a park can help cities improve their quality of life [38]. Although park use was not directly related to biodiversity, visitors perceived parks as locations where people could engage with nature in a variety of ways [28]. Plant, especially trees, has an impact on thermal comfort, wind speed, and air pollution and increases the leisure space for occupants. A low ecological landscape is preferable when trees are present due to a lower blocking effect on wind and pollutant dispersion [39].

An urban park should have natural components and a range of floral species, while different groups value different amenities. These insights may aid park designers and policymakers [11]. Urban parks are important biodiverse hotspots and part of urban green infrastructure. Numerous factors influence urban park biodiversity, vegetation layout, and ecological function [36]. Assessing the ecosystem service benefits of urban parks can help maintain and manage urban green spaces [40].

During the lockdown and limits on public activities and meetings, green spaces have emerged as a source of resilience in the midst of the coronavirus epidemic, partly due to their beneficial impacts on psychological, physical, and social cohesiveness as spiritual wellness [41]. As the frequency of older persons with disabilities increases, urban parks must be accessible to this demographic to benefit from the same health benefits as the general population [19].

To maximize parks’ public health advantages and make them welcoming and accessible to users of all ages, cultures, and abilities, meaningful collaboration between park planners, municipal governments, and persons with disabilities is essential. Co-designing parks with individuals with disabilities may be one way to increase park accessibility and usage and, consequently, park participation for older persons with disabilities [19]. City parks should provide appropriate activities to promote restorative environments, particularly for subjects with a high degree of education and stress [42]. Suitable microclimate design for urban parks is critical since it affects thermal comfort and thus the public’s use of outdoor areas. Modifying the vegetation arrangement can benefit the microclimate, particularly during the hot season [31].

The research revealed that the park inside the study area has developed into a significant component of the open green space. The presence of plants defines the vegetation density class in the park. Due to the park’s composition and arrangement inside the park regions, the urban park demonstrates several classes. The component relates to the park’s function of promoting the environment’s and people’s quality of life as public areas.

The findings may demonstrate the importance of green open spaces, especially parks, in enhancing the quality of life in urban areas. The presence of trees and other plants in the park affects the area’s surface temperature. Each park’s unique qualities result in a distinct class of vegetation density and surface temperature. In carrying out the park’s role, the manager divided the space for relaxation and sport, resulting in the formation of an open area with less vegetation. Thus, the park’s area is divided into dense vegetation areas dominated by trees and more open areas for functional services. To maximize the park’s function, particularly in contributing to the quality of green open space, the manager should develop a plan after intensive study. The study includes the arrangement of green space and socio-service areas while considering the park’s ecological purpose and sustaining the park’s environmental quality.

## 5. Conclusions

Most urban areas were classed as having a low density, as measured by NDVI. The NDBI values were higher in most of the areas. This means that the vast majority of places have less vegetation. The research reveals the area’s mix of density classes in terms of parks. All parks were categorized in all vegetation density classes. This is because of the location of park components such as trees, mixed plant communities, recreation, sporting spaces, etc. The LST analysis indicates that the temperature in the urban park was lower than in the surrounding areas. The area close to the urban park is considerably cooler than the area further away from the park. The presence of trees was the most component affecting the surface temperature. The clear proof can be seen in all parks except Merdeka Square Park. The park has large bare areas, and trees are mostly in the outer part of the main field. Ahmad Yani Park shows the lowest temperature due to the densest trees growing within the park. It is critical to maximizing the function of parks as green open spaces, particularly in Medan. Parks must be upgraded in terms of vegetation and infrastructure as a crucial component of metropolitan regions. The park’s administration might increase the vegetation to optimize the land and maintain the park to improve the city’s quality of life.

## Figures and Tables

**Table 1:** Description of Parks.

Name of Park	Area (m^2^)	Location (Sub-District)
Medan Merdeka Square	48,000	Medan Barat
Ahmad Yani	15,200	Medan Maimun
Teladan	15,500	Medan Kota
Sri Deli	13,159	Medan Kota
Gajah Mada	7008	Medan Baru
Petula	4194	Medan Baru
